# Kinematic characteristics of lumbar spinous processes during axial rotation in patients with lumbar degenerative disc disease lateral lumbar interbody fusion and intervention

**DOI:** 10.1186/s12891-017-1504-6

**Published:** 2017-04-04

**Authors:** BaoLiang Zhang, Wenhan Huang, Hong Xia, Xinglong Feng

**Affiliations:** 1 Department of Orthopedics, The Central Hospital of Yongzhou, Hunan, China; 2 Department of Orthopedics, Guangzhou General Hospital of Guangzhou Military Command, Affiliated of Southern Medical University, 111 Liu-hua Avenue, P.O. Box 510010, Guangzhou, China; 3 Department of Orthopaedics and Traumatology, Prince of Wales Hospital, The Chinese University of Hong Kong, Hong Kong, China; 4 Southern Medical University, 1838 North Guangzhou Avenue, P.O. Box 510515, Guangzhou, China; 5 Guangzhou Panyu Central Hospital, Guangzhou, China

## Abstract

**Background:**

Data about minimally invasive surgery for the treatment of patients with degenerative disc disease (DDD) has been reported. However, no quantitative knowledge about the biomechanical characteristics of the spinous processes in patients with DDD after operation was reported in the literature.

**Methods:**

Fourteen adult patients with DDD at the L3-4 level were recruited and scanned using computed tomography (CT) to construct three-dimensional (3D) anatomical vertebral models of L2-5. These patients were asked to maintain four positions to acquire 6DOF data about the area of the spine being investigated (L2-5). Fluoroscopy was used to capture spinal motion. 6DOF data from the fluoroscopic images of the four positions was compared to evaluate the kinematics before operation and 6 months after direct lateral interbody fixation (DLIF).

**Results:**

Altered kinematics were found mainly in rotation. For the images captured while patients were in the supine position, no significant differences were detected in different functional positions before and after operation. At other positions, the most kinematic involved level was the L3-4 level, which was followed by the L4-5 level. The range of flexion-extension motion at all levels decreased after operation (by an average of 1° to 7°) while different trends were found in left-right bending/rotation. Overall, after surgical treatment, functional activities were partially restored.

**Conclusions:**

Overall the lumbar spinous processes (LSP) at each level responded differently, regarding rotation, before and after DLIF. This data provides new insights for the evaluation of function before and after surgical treatment in patients with LSP disease.

## Background

The lumbar spinous processes (LSP) is very crucial to the protection of the neural structure in the spinal cavity and the stabilization of the segmental spine unit. Xia et al. [1] provide kinematic analysis of the LSP in asymptomatic patients when performing unrestricted functional body motions. For pathological conditions, minimally invasive surgery will be performed to treat patients with DDD [2]. However, no quantitative data associated with biomechanical characteristics about spinous processes from patients who have undergone a spinal operation is reported in the literature. This knowledge is of great importance to the evaluation of the surgical treatment’s efficacy, including lumbar discectomy and fusion for lumbar disk disease.

Some studies report on the movement features of the face joints, vertebral bodies, and intervertebral discs [3–5]. Data about the movement features of the LSP was investigated by using anteroposterior radiographs [6], cadaveric specimens [7], and CT [8]. However, no researchers review the movement and biomechanical features of the LSPs in patients before and after surgical treatment.

In current study, the 6° of freedom (6DOF) of LSP from L2 to L5 in DDD patients will be determined in the following four postures: supine, vertical standing, maximum trunk flexion and maximum trunk extension before and after direct lateral interbody fixation (DLIF) by using a single fluoroscopic imaging system. We hypothesized that after DLIF, the LSP at the DDD levels would have limited motion and that adjacent levels would demonstrate increased motion when compared to the same positions on the same patients before operation.

## Methods

### Patient samples

Seven male patients and seven female patients with DDD between L3 and L4 (57.6 ± 4.8 years; BMI 24.1 ± 3.7 Kg/m^2^) were recruited from a single academic center. The disc degeneration of each patient was classified using Pfirmann classification based on clinical radiographic assessments [9] (Table 1). Before launching the study, Research Ethics Board at Guangzhou General Hospital of Guangzhou Military Command approved the study. Each patient signed a consent form before the study.

**Table 1:** Disc degeneration graded with P firmann system for DDD patients

Subject	#1	#2	#3	#4	#5	#6	#7	#8	#9	#10	#11	#12	#13	#14
Disc degeneration grade	2	4	2	3	3	4	3	3	4	4	2	3	4	2

### Imaging technique

**Fig. 1:**
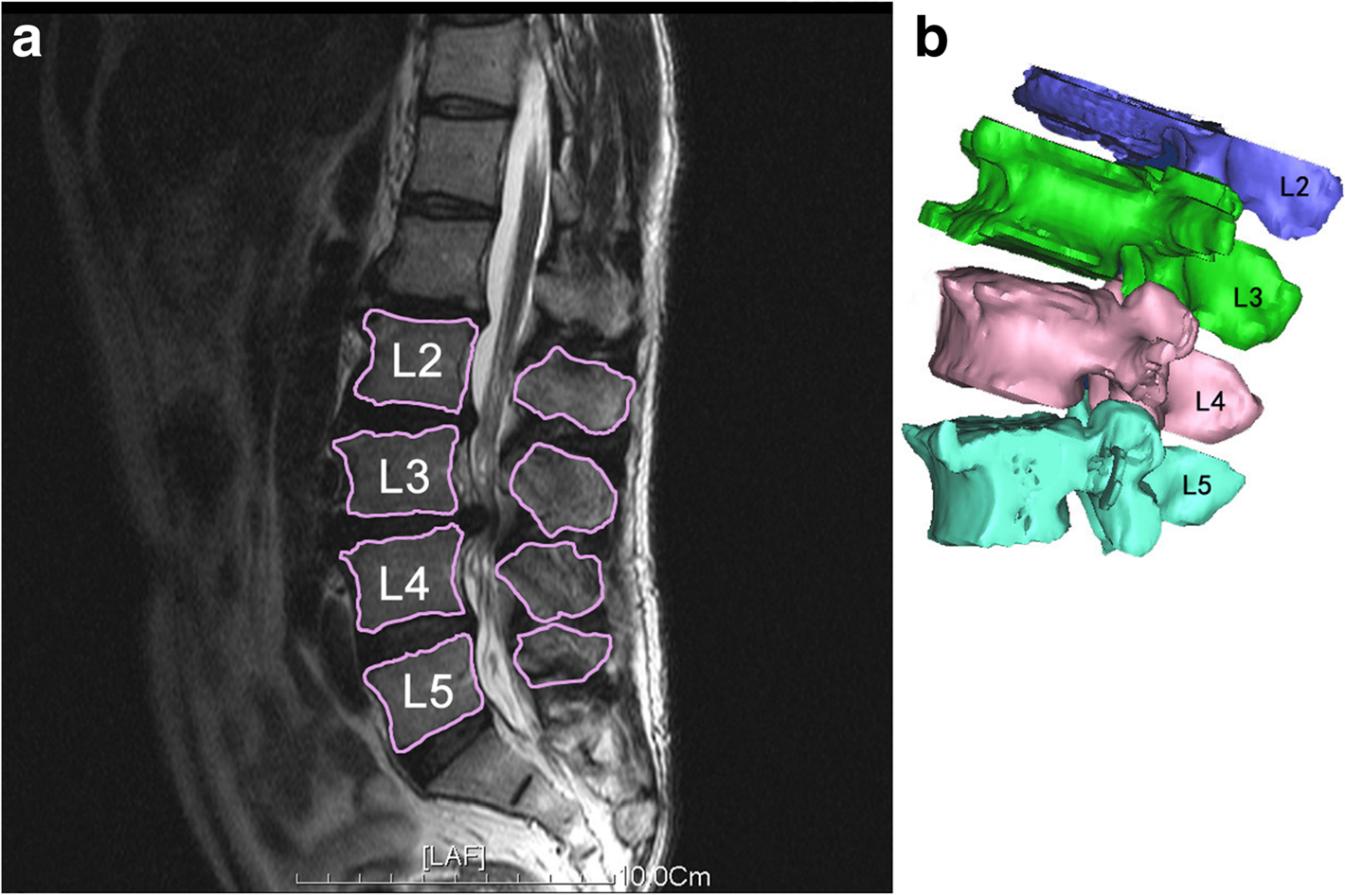
**a** MR image of human lumbar spine of DDD patients. **b** 3D anatomical vertebral model from L2 to L5 constructed using the MR images. Local coordinate systems at the tip of spinous process were used to calculate the relative 6DOF kinematics of the proximal spinous process with respect to distal spinous process

**Fig. 2:**
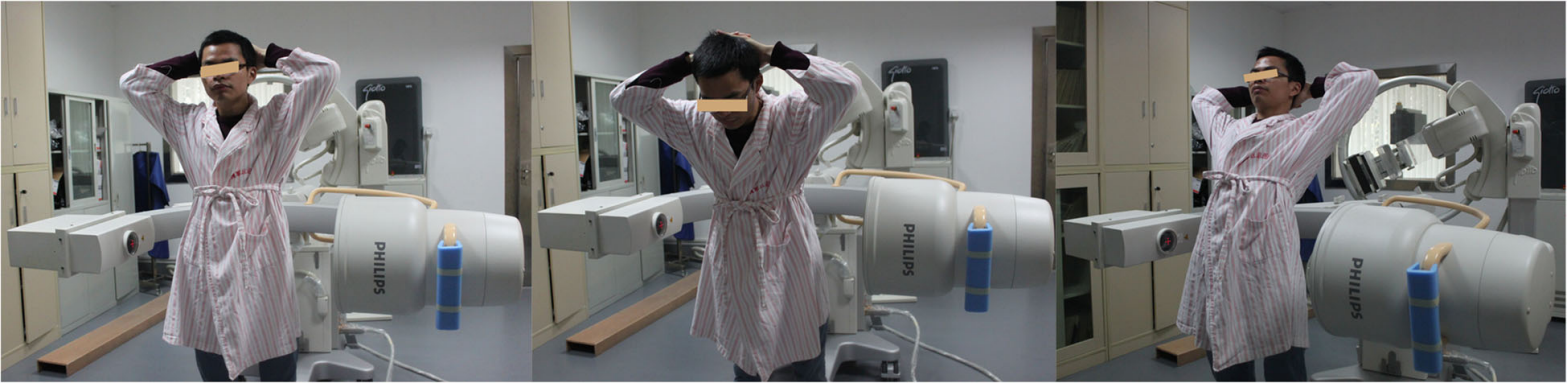
A single fluoroscopic imaging system for standing, flexion and extension positions in living subjects

To construct the 3D vertebral models, CT scanning of a portion of each subject’s spine (L2-L5) was performed. The CT files in Digital Imaging and Communications in Medicine (DICOM) format of the spinal segments were then processed in an image processing software (Mimics 17.0, Leuven, Belgium) to create 3D vertebral models of L2-L5 using a protocol verified by Li et al. [10]. Mesh models of the vertebrae and lumbar spinous processes were derived from bony outline (Fig. 1a and b). Next, each patient’s lumbar spine was dynamically imaged using a single fluoroscopic imaging system while patient maintained different weight-bearing positions: supine, standing, maximal flexion, and maximal extension (Fig. 2). The subjects were required to sustain each position for approximately 1 s, meanwhile the fluoroscope dynamically scanned sections of their spines in a lateral direction. The real-time positions of the vertebrae were constructed in the Mimics software using the solid models and the corresponding dynamic lumbar spine images [10]. The 3D vertebra models can be individually rotated and translated in 6DOF. A 2D-3D matching was achieved by matching model projections and the osseous outlines from the fluoroscopic system. Therefore, we could determine the positions of specific vertebrae and spinous processes for each pose. In a similar study by Yao et al. [11], the accuracy of this technique was within 0.6 mm for translation and 1.3° for rotation.

### Surgical procedure

**Fig. 3:**
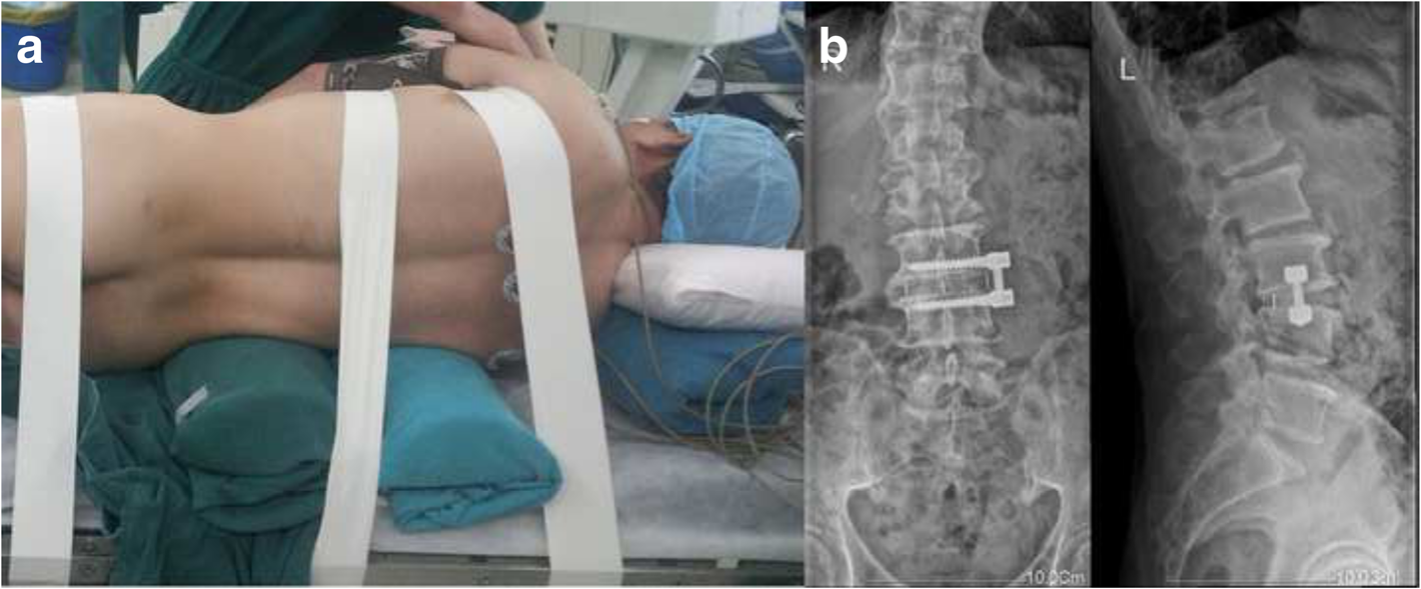
**a** Lateral incision. **b** Anterior-posterior and lateral X-ray image of DDD patients showing lateral trans-psoas interbody fusion at the L3-4 level with a side plate and interbody fusion mass as depicted by white markers

After general endotracheal anesthesia, patients were put in lateral positions (Fig. 3a). The target level was determined through C-arm fluoroscopy after disinfection and draping. A skin incision of approximately 3 cm in length was made at the marked sites. Afterwards, the retroperitoneal fat was exposed, the psoas muscle was dissected, and the tubular retractor was fixed. This was followed by the excision of the intervertebral disc and endplate preparation. Next, the fusion materials were inserted into a cage. Then we used the demineralized bone matrix (DBM) or tricalcium phosphate (TCP) for bone fusion. We used Osteofil (Medtronic, Memphis, TN, USA) as the graft material (Fig. 3b).

### Measurement of the kinematics in spinous processes

**Fig. 4:**
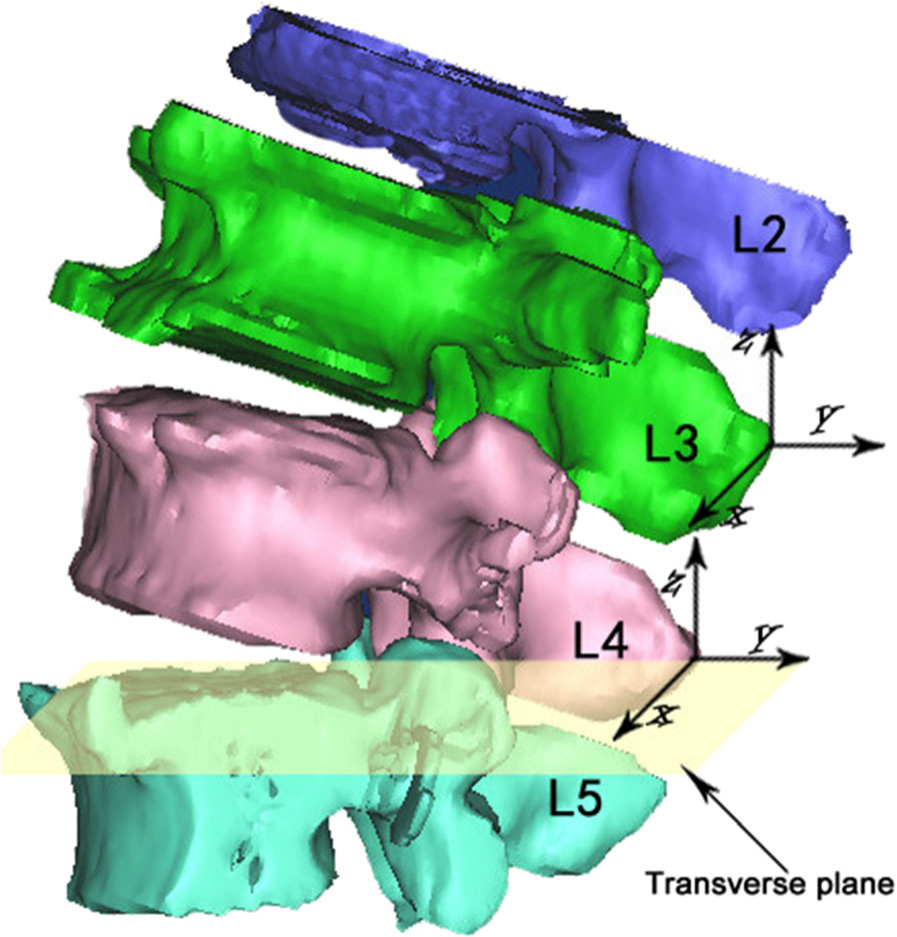
Local coordinate systems were established at the tip of the process to measure 6DOF flexion(+)/extension(-); left bending(+)/right bending(-); left rotation(+)/right rotation(-); left translation(+)/right translation(-); posterior translation(+)/anterior translation(-); proximal translation(+)/distal translation(-);a degree (°) for rotation; b mm for translation

After obtaining the real-time 3D positions of the vertebrae and spinous processes, kinematic analysis was performed using customized software (Matlab 7.0, the Mathworks, Inc.) [12]. In the current study, the kinematics of adjacent levels (L2-L3, L3-L4, and L4-L5) were determined when the subjects sustained the following four postures: supine, standing, maximum trunk flexion, and maximum trunk extension. Specifically, the kinematics were determined based on the relation of the inferior spinous process to the superior spinous process. A Cartesian coordinate system was established to describe the tip point positions of the spinous processes in 3D space (Fig. 4). The x-axis is perpendicular to the sagittal plane and points left, the y-axis is parallel to the sagittal plane and points in the posterior direction, and the z-axis is perpendicular to the x and y planes and points cranially. In addition, we investigated the range of motion (ROM) in flexion-extension, left-right bending, and twisting at each vertebral level. In addition, the raw data of the current study would be uploaded to figshare (https://figshare.com/articles/Data_BMSD_D_16_00686_xlsx/4757119) as a supplementary file.

### Statistical analysis

A paired *t*-test was used to compare the kinematic changes at the L2-L3, L3-L4, and L4-L5 spinous processes in these four positions. The level of significance was set to *p* <0.05. SPSS 13.0 for Windows (StatSoft version 8.0, Tulsa, Ok, USA) was used to perform statistical analysis.

## Results

### Supine position

#### Rotation kinematics

**Fig. 5:**
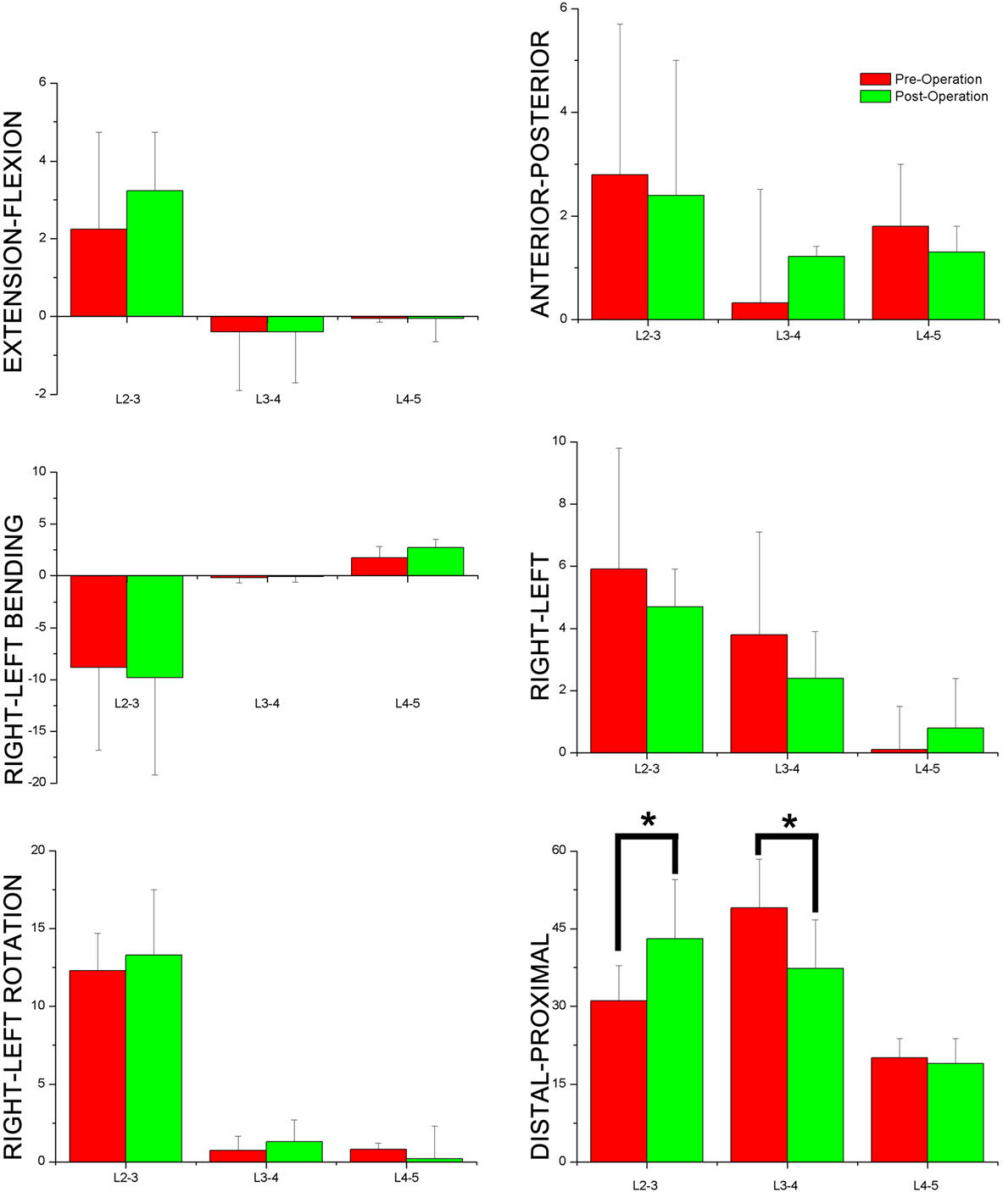
6DOF of lumbar spinous process during supine position flexion(+)/extension(-); left bending(+)/right bending(-); left rotation(+)/right rotation(-); left translation(+)/right translation(-); posterior translation(+)/anterior translation(-); proximal translation(+)/distal translation(-) a degree (°) for rotation; b mm for translation

No statistical differences were found regarding rotation in the supine position in the two groups (Fig. 5). In terms of flexion/extension, the ROM is small (average 0° to 3°).

#### Translation kinematics

With respect to translations in the frontal plane, the ROM is small (on average 0-6 mm). In the context of proximal/distal translation, the ROM is larger compared to other translation freedoms (19 mm to 49 mm on average). After operation, L2-3 show more distal translation (31.1 ± 6.8 mm vs. 43.1 ± 11.4 mm, *P* <0.01). In contrast, L3-4 in the postoperative group show reduced distal translation (49.1 ± 9.4 mm vs. 37.3 ± 10.4 mm, *P* < 0.01).

### Standing position

#### Rotation kinematics

**Fig. 6:**
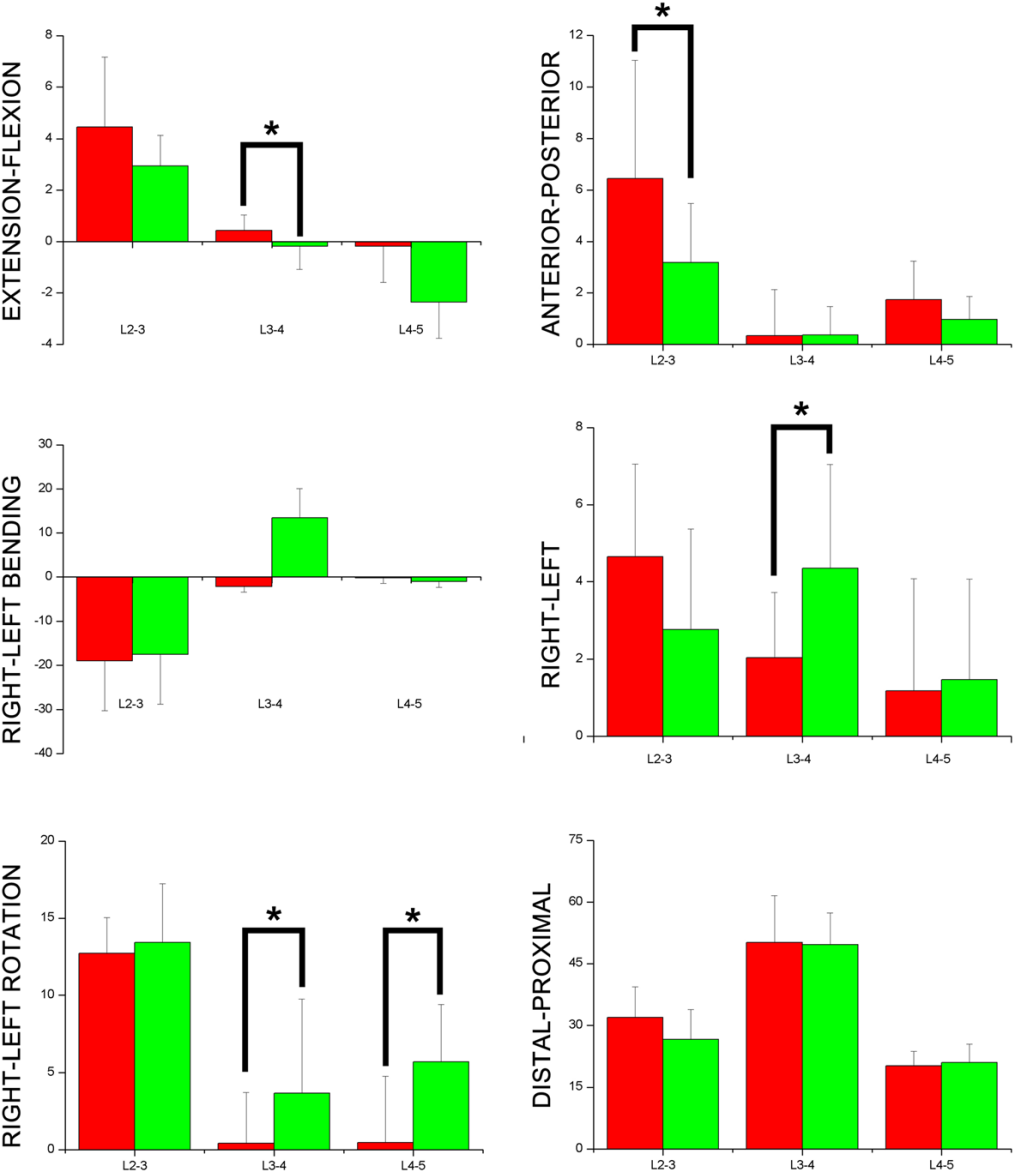
6DOF of lumbar spinous process during standing position flexion(+)/extension(-); left bending(+)/right bending(-); left rotation(+)/right rotation(-); left translation(+)/right translation(-); posterior translation(+)/anterior translation(-); proximal translation(+)/distal translation(-) a degree (°) for rotation; b mm for translation

The spinous processes at different vertebral levels have different patterns of kinematics in functional weight-bearing positions (Fig. 6). In the standing position, with respect to flexion/extension, spinous processes at L4-5 exhibited 0.18 ± 1.4° of extension before operation and 2.3 ± 1.4° of extension after operation (P =0.041). Regarding twisting, both postoperative groups in L3-4 levels and L4-5 levels show increased left twisting (0.4 ± 3.3° VS. 3.6 ± 6.1°, P < 0.01 and 0.5 ± 4.3 VS. 5.7 ± 3.7°, *P* <0.01).

#### Translation kinematics

Significant differences were found in left-right and anterior-posterior translation. L3-4 in the postoperative group show increased left translations (2.0 ± 1.7 mm vs. 4.3 ± 2.7 mm, *P* = 0.033). With respect to anterior-posterior translation, L2-3 in the postoperative group showed decreased anterior translations (6.4 ± 4.6 mm vs. 3.2 ± 2.3 mm, *P* = 0.047).

### Maximum flexion position

#### Rotation kinematics

**Fig. 7:**
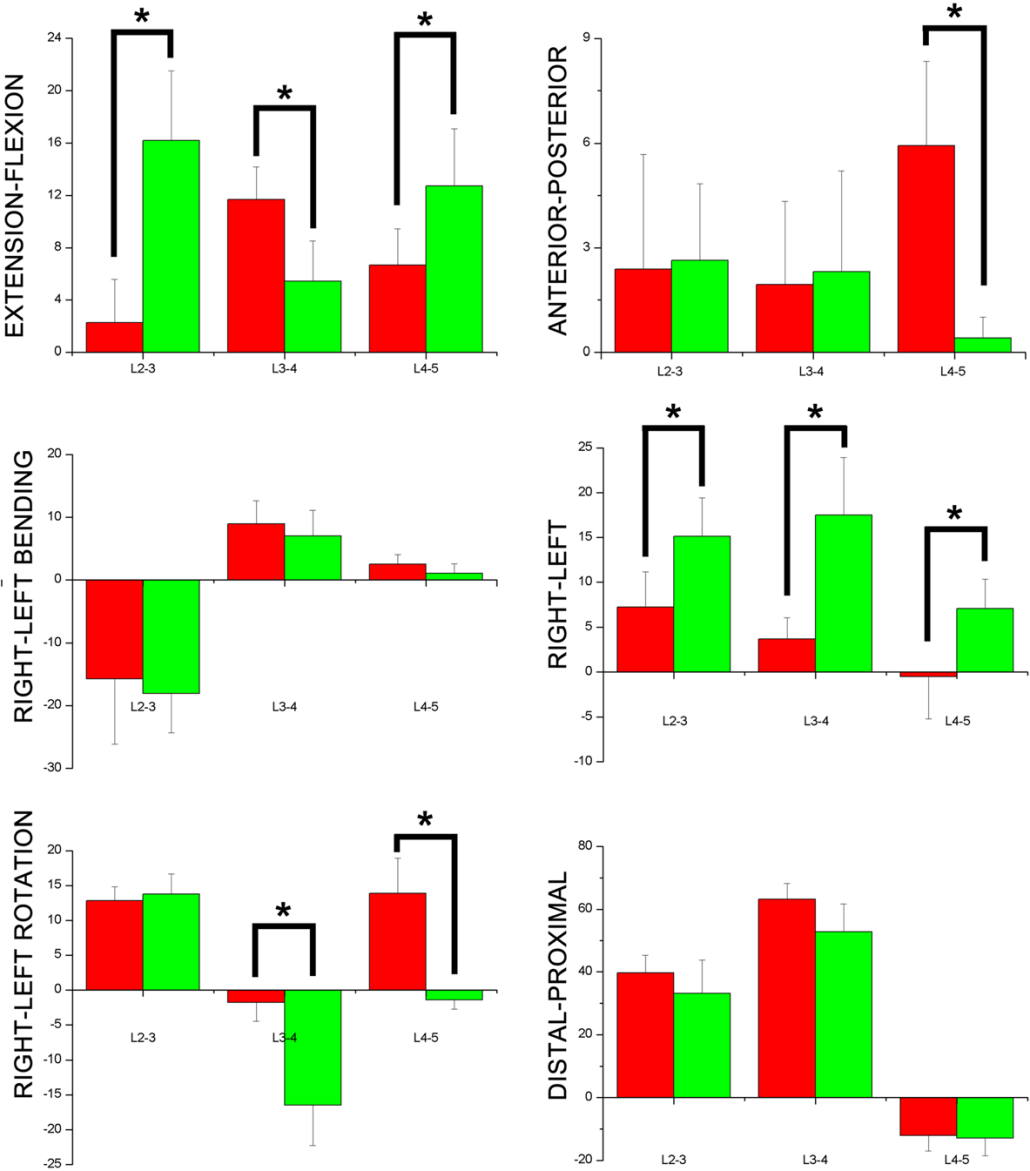
6DOF of lumbar spinous process during flexion position flexion(+)/extension(-); left bending(+)/right bending(-); left rotation(+)/right rotation(-); left translation(+)/right translation(-); posterior translation(+)/anterior translation(-); proximal translation(+)/distal translation(-) a degree (°) for rotation; b mm for translation

Different levels exhibited distinct patterns of rotation. With respect to flexion/extension (Fig. 7), with the exception of L3-4, the other two levels show increased flexion. L3-4 exhibited less flexion than pre-operation (11.7 ± 2.5° vs. 5.4 ± 3.1°. *P* = 0.028) while L4-5 showed increased flexion compared to preoperative figures (6.7 ± 2.8° vs. 12.7 ± 4.4°. *P* <0.01), and L2-3 showed increased flexion (2.3 ± 3.3° vs. 16.2 ± 5.3°, *P* = 0.014). For rotation in transverse plane, both L3-4 and L4-5 showed an increase in right rotation (1.8 ± 2.7° vs. *16*.5 ± 5.8°, *P* <0.01, and 13.8 ± 5.1° vs.*-1.5* ± 1.3°, *P* <0.01, respectively)

#### Translation kinematics

In left/right translation, all levels show an increase in left translation (15.1 ± 4.3 mm, 17.5 ± 6.4 mm, 7.1 ± 3.2 mm, respectively for L2-3, L3-4, and L4-5). In the context of anterior/posterior translation, L4-5 exhibited a sharp decrease in anterior translation (5.9 ± 2.4 mm vs. 0.5 ± 0.6 mm, P = 0.03).

### Maximum extension position

#### Rotation kinematics

**Fig. 8:**
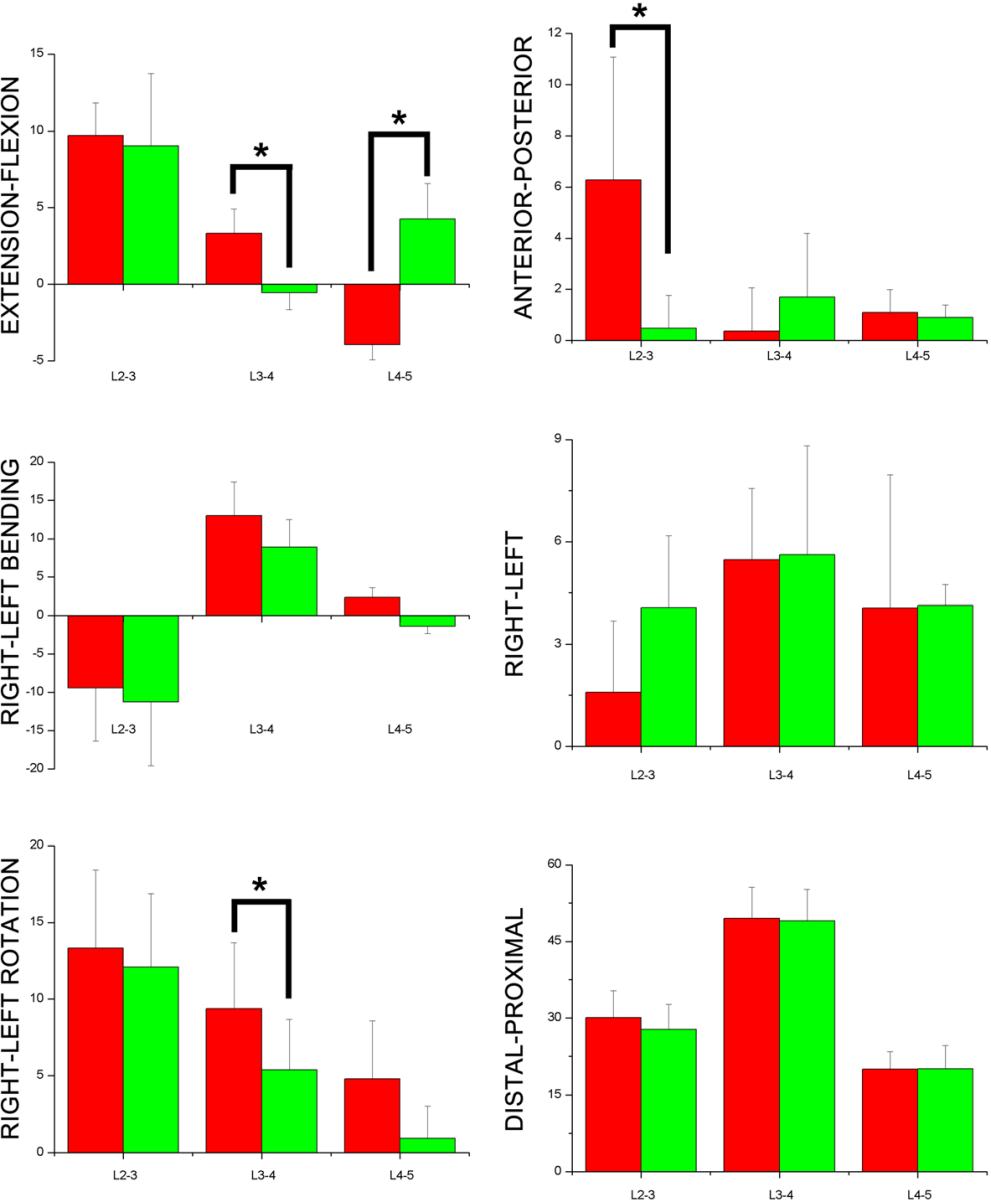
6DOF of lumbar spinous process during extension position flexion(+)/extension(-); left bending(+)/right bending(-); left rotation(+)/right rotation(-); left translation(+)/right translation(-); posterior translation(+)/anterior translation(-); proximal translation(+)/distal translation(-) a degree (°) for rotation; b mm for translation

In flexion/extension, for L4-5, increased extension was exhibited (3.9 ± 1.1° vs. 4.3 ± 2.3°. *P* <0.01) (Fig. 8). However, for L3-4, a decrease in the flexion angle was shown (-0.6 ± 1.1°). In left/right rotation, L3-4 showed decreased left rotation (9.3 ± 4.3° vs. 5.4 ± 3.3°. *P* = 0.02).

#### Translation kinematics

Statistical differences were only found in anterior-posterior translation. Anterior translation levels for L2-3 showed a sharp decrease of after operation (6.3 ± 4.8 mm vs. 0.9 ± 0.5 mm, *P* <0.01).

## Discussion

In the current study, we measured the changes of kinematics in spinous processes in patients before and after DLIF at different body positions in axial rotation. We found that, in general, altered kinematics mainly took place in rotation. In the supine position, significant differences were mainly detected in proximal-distal and left-right translation before and after operation. In the standing position, L4-5 exhibited increased extension and left rotation compared to those from before the operation. In the flexion/extension posture, alterations in 6 DOF of the inter-spinous processes (ISP) showed different trends in different DDD patients. Generally, these changes imply that disc degeneration and fusion intervention distinctly correlate to alterations in ISP motions at involved levels [13].

Only several studies report on the 3D kinematics of the LSP, especially regarding pathological and intervention conditions, such as artificial disc replacement or lumbar discectomy followed by fusion [6, 8, 14]. Ihm et al. investigated the lumbar ISP distance and demonstrated a declining trend regarding the ISP distance along with increased age [15]. Sobottke et al. measured the anatomical features of inter-spinous space and spinous processes. They reported that the anterior position is a optimum choice for a stand-alone inter-spinous spacer [8]. However, the 2D classification methods led to increased result variability due to the limited and inexact identification of the identical anatomic landmarks [16, 17]. The 2D-3D registration method is a more accurate approach compared to 2D classification methods [18]. Xia et al. investigated the 3D motion characteristics of ISP distances in healthy subjects and found that changes in ISP distances are positively correlated to vertebral levels and body postures [1]. Yao et al. reported that, in supine, standing, and extension positions, ISP were physically smaller in patients with DDD than healthy subjects. [11]. The repeatability is 0.3 mm for translation and 0.6° for rotation. However, we can speculate that ISP distances cannot exactly define the tracking of ISP because ISPD is an indirect measurement method with which to explore ISP kinematics. In the study by Xia et al. [1], ISPD is defined as the shortest distance between the lowest tip of the inferior spinous process and the highest tip of the superior spinous process. To some extent, it represents the partial change of adjacent joints between spinous processes as only vertical translation was described in this method. Movement in other directions was not evaluated.

No researchers have performed kinematic analysis of spinous processes in patients who have DDD during in vivo weight-bearing movements. Quantitative knowledge about adjacent spinous processes is very important to understanding spinal pathology. It is also very critical to improve the current surgical treatment approaches for spinal diseases. In recent years, it has become very popular to measure the kinematics (including 6DOF and ROM) in adjacent joints, such as knee joints and lumbar inter-vertebrae, using fluoroscopic technology [10, 19].

### Rotation

In our study, we found that ROM in flexion/extension rotation was smaller in post-operation patients at all investigated levels when compared to pre-operation, which is likely to increase the risk of adjacent segmental disorders. Decreased ROM happened in trunk standing, flexion, and extension positions. One of the major disadvantages of anterior lumbar interbody infusion (ALIF) is that it demonstrated abnormal kinematics of the spine, and therefore, may lead to degeneration at adjacent segments. These abnormal alterations potentially require additional fusion. Other potential complications include ileus, vascular injury, and retrograde ejaculation. Thus, in recent years, a lateral trans-psoas approach (DLIF) has been utilized to prevent from the limitations at the involved levels and allows patients to quickly return to routine activities [20]. In addition, this method can improve spinal instabilities as well as deformities and avoids to perform a posterior approach. Here, we detect alterations in kinematics after DLIF. Except for sagittal rotation, we also found that ROM in left/right bending and transverse rotation decreased. We speculate that this is due to the enhanced tissue strain, facet joint pressures, and increased intradiscal at the levels that are adjacent to the fusion. This motion data concurs with our ISP kinematic alterations between preoperative and postoperative groups.

### Translation

Statistical differences were mainly detected in the trunk flexion position. In this position, LSPs at all levels exhibited statistical differences in left/right translation. We found increased left translation in all three ISP. Besides, significant differences were found in left/right rotation at all three levels. Yao et al. reported that, in non-weight-bearing supine, standing, and extension positions, ISP were physically smaller in patients with DDD than healthy subjects [11]. In the current study, similar trends were exhibited in all groups. Cinotti et al. reported that, compared healthy intervertebral discs, degenerated intervertebral discs result in the reduced height of posterior structures [20]. DLIF has the advantage of protecting the facet joint, the posterior longitudinal ligament, and the anterior longitudinal ligament, which enables it to improve spinal alignment and stabilization. Therefore, it is likely to replace conventional options of lumbar interbody fusion, like ALIF, as an alternative surgical approach [21]. DLIF is able to provide strong mechanical stability via a large interbody constructure and the sparing of ligamentous structures [22]. In our data, these different patterns of kinematic alteration imply that after DLIF, different strategies were shown in different vertebral spinous processes from dysfunctional to re-stabilization conditions.

The results of the current study have some clinical significance regarding the interpretation of LSP in the pathogenesis of low back pain that is not clear. Our study indicates that, for patients with DDD, the ROM of the ISP is reduced during in vivo functional movements. Lately, ISPD has been used as an alternative surgical approach to conventional decompressive surgery for the treatment of DDD and favorable clinical outcomes have been shown [23, 24]. However, some complications with the use of inter-spinous implants have been reported, such as implant movement [23–25]. Kim et al. argued that after inter-spinous process spacer (IPS) surgery, degenerative lumbar spondylolisthesis is strongly correlated with the prevalence of spinous process fracture [23]. In the current study, we found that the ROM of ISP change increased for two postures (maximum flexion and extension) after operation. This implies that, after the introduction of inter-spinous implants in DDD patients, hypermobility should be restricted to sustain the position of a loosely-fitted device in case the ISP dislocates. The kinematic characteristics of LSPs provide some biomechanical information that can help with decisions about the use of compressible or rigid devices.

## Limitations

This study has some limitations. The patients included in the current study were specifically recruited because they had DDD at between L3 and L4. Therefore, they only represent a certain percentage of all DDD patients. It is reasonable to believe that kinematic patterns may differ in patients with various disc degenerative patterns. Patients with DDD at diverse segments of the spine should be included in further studies. A small number of subjects was included because it was difficult to find patients that met the inclusion criteria. The process used to attain scientific 3D kinematic data was highly time consuming, which reduced efficiency.

## Conclusion

In conclusion, our study provides quantitative data about the 6DOF of the LSP during supine, standing, maximum trunk flexion, and maximum trunk extension positions in patients with DDD before and after DLIF. It is of great importance to the evaluation of the efficacy of DLIF. The findings imply that disc degeneration and fusion intervention correlate with distinct kinematic alterations of the ISP at the involved level.

## Abbreviations

6DOF: 6° of freedom
DDD: Degenerative disc disease
DLIF: Direct lateral lumbar interbody fusion
LSP: Lumbar spinous process
